# The impact of the euthanasia assessment procedure: a qualitative interview study among adults with psychiatric conditions

**DOI:** 10.1186/s12888-022-04039-2

**Published:** 2022-06-27

**Authors:** Monica Verhofstadt, Kenneth Chambaere, Koen Pardon, Freddy Mortier, Axel Liégeois, Luc Deliens, Kurt Audenaert

**Affiliations:** 1 End-of-Life Care Research Group, Department Public Health and Primary Care, Vrije Universiteit Brussel (VUB) & Ghent University, Corneel Heymanslaan 10, 6K3 Ghent, Brussels Belgium; 2 Bioethics Institute Ghent, Ghent University, Ghent, Belgium; 3 Faculty of Theology and Religious Studies, KU Leuven, Louvain, Belgium; 4 Organisation Brothers of Charity, Ghent, Belgium; 5 Department of Psychiatry, Ghent University Hospital, Ghent, Belgium

**Keywords:** Euthanasia, Mental disorders, End-of-life decisions, Assisted suicide

## Abstract

**Background:**

Assisted dying for adults with psychiatric conditions (APC) is highly controversial but legally possible in a few countries, including Belgium. Previous research has suggested that the complex euthanasia assessment procedure may cause additional suffering in APC but may also induce positive experiences. This study reports on the impact of the euthanasia assessment procedure as experienced by APC on three counts: 1) their mental state, including death ideation; 2) their treatment trajectory; 3) their social relationships.

**Methods:**

We performed an in-depth qualitative interview study with 16 APC in Flanders, Belgium, who had voiced a euthanasia request between 2016–2020. Thematic coding was used.

**Findings:**

We interviewed 16 APC. Euthanasia assessment procedures brought out a plethora of experiences in APC, both favourable and unfavourable. Whereas thoughts of suicide remain present to a certain extent, being in the assessment procedure allows some APC to reconsider alternatives towards life, and also to attempt new treatment options. However, many APC experience ambivalence about the supposedly inherent desirability and dignity in euthanasia. Worries also surfaced about the rationale behind and effects of involvement of APCs’ social circle, and about the impact it could have on them.

**Conclusion:**

Further research, including other stakeholder perspectives, is recommended with a view to maximising favourable and minimising unfavourable impacts for all involved. In clinical practice attention to these impacts is paramount, and clear communication and management of expectations between physician and patient, seems appropriate to address the many ambivalent experiences that accompany APC during the euthanasia assessment procedure. Policy attention could in this regard go to clarifying certain sources of ambivalence and issues that are insufficiently addressed, such as modalities of relatives’ involvement.

**Supplementary Information:**

The online version contains supplementary material available at 10.1186/s12888-022-04039-2.

## Introduction

Assisted dying, defined as the act to end life by providing, prescribing or administering lethal medication at the competent patient’s explicit request, is – under certain conditions (see Table 1) – legal in an increasing number of countries around the globe [1]. Euthanasia’ refers to the act of a physician administering the lethal medication; ‘assisted suicide’ refers to the act of prescribing or providing the medication to the patient, who then self-administers it. Belgium is one of the earliest countries to enact euthanasia legislation [2]. The Netherlands (2002) [3], Belgium (2002) [2], Luxembourg (2009) [4] and Spain (2021) [5] are the sole countries in the world to enact legislation that does not rule out adults with psychiatric conditions (APC) as sole or primary underlying conditions. Canada is currently also considering expanding current legislation to APC [6, 7]. Although euthanasia (the act of a physician administering the lethal medication) is legalised and implemented in Belgium for almost two decades, it remains highly controversial when applied in APC.

**Table 1:** Due care criteria for euthanasia for psychological suffering (Belgian Law on Euthanasia 2002)

Substantive criteria
Euthanasia for psychological suffering is allowed if the patient: 1. is an adult (> 18 years old) or emancipated minor; 2. is legally competent and conscious at the moment of the euthanasia request; 3. has made a voluntary, well-considered, and repeated request, that is not the result of any external pressure; and 4. is in a medical situation, without prospect of improvement, of constant and unbearable psychological suffering a) that cannot be alleviated; and b) that results from a serious and incurable condition caused by accident or illness
Procedural criteria
Administrative requirements
1. The patient’s request must be in writing and drawn up, dated and signed by the patient. If the patient is not physically capable of doing so, the document should be drawn up by an adult designated by the patient and without material interest in the death of the patient. In such case, the request is drafted in the presence of a physician whose name is recorded in the document 2. At least one month should proceed between the patient’s written request and the performance of euthanasia 3. The patient may revoke the euthanasia request at any time, in which case the document is removed from the medical record and returned to the patient 4. All the requests formulated by the patient, as well as any actions by the attending physician and their results, including the reports of the consulted physicians, are noted in the patient’s medical record 5. Within four working days after performing the euthanasia, the attending physician is required to complete the registration form and to deliver this document to the Federal Control and Evaluation Commission for Euthanasia, so as to allow the Commission to determine whether the euthanasia was performed in accordance with the legal due care criteria
Decision-making procedure
The attending physician must: 1. inform the patient about her health condition and life expectancy; 2. discuss with the patient her euthanasia request and any therapeutic and palliative options still remaining and their consequences; 3. be certain that all substantive criteria have been met, including the patient’s constant and unbearable suffering that cannot be alleviated and the durable nature of the request, and to this end; A. have several conversations with the patient, spread out over a reasonable period of time, taking into account the progress of the patient’s condition; B. consult a second physician, i. who must be independent and competent to give an opinion on the condition concerned; ii. who must review the medical record and examine the patient; and iii. who must ascertain the patient’s constant and unbearable suffering that cannot be alleviated; C. consult a third physician, i. who must be independent and a psychiatrist; ii. who must review the medical record and examine the patient; and iii. who must ascertain the constant and unbearable suffering that cannot be alleviated, and the voluntary, well-considered, and repeated nature of the euthanasia request; d. if there is a nursing team that has regular contact with the patient, discuss the request with that team or with members of that team; e. if the patient so desires, discuss the request with the relatives appointed by the patient; and f. ascertain that the patient has had the opportunity to discuss the request with the persons whom she designates

Since legalisation, 315 cases of euthanasia in APC have been carried out in Belgium, 1.4% of all reported *performed* euthanasia cases [8–11]. This is only a proportion of all APC *applying*for euthanasia, as a recent annual report from one Belgian end-of-life consultation centre revealed that 12% of euthanasia requests by APC lead to euthanasia [12], a.o. around half of them putting their request on hold or dying through suicide or palliative sedation (1%) [12]. The reasons for these outcomes are largely unknown, but as scarce research shows, these might be related in large part to the often high complexity of APCs’ life and treatment histories, as well as continued controversy about assisted dying in this patient group, and perhaps also ambivalent feelings about the wish to die in APC themselves [13–15].

Several mental health organisations have recently published advisory texts [16, 17], in which existing legal criteria are ‘operationalised’ strictly and a number of further due care criteria expressed for APC. For instance, the criterion incurability of the disorder is defined as ‘no reasonable treatment perspective’ and a number of clinical conditions that needs to be met were set. Emphasis is also put on the importance of extending the one-month reflection period either to 6 months or one year, of engaging a minimum of two advising psychiatrists (instead of one), and of engaging APC’s other relevant caregivers and social inner circle in euthanasia assessment procedures. A recent survey among Belgian psychiatrists showed that these additional due care criteria are already implemented in practice, with the entire assessment procedure of requests culminating in the performance of euthanasia spanning on average 13.5 months, and encompassing multidisciplinary consultations, including with family and friends [18].

Two qualitative studies revealed that some APC experienced the euthanasia procedure itself as a cause of additional suffering, while to other APC it may offer the needed support to find new perspectives on life [19, 20]. However, these studies lacked an in-depth focus on the impact of the euthanasia assessment procedure. In addition, a survey among psychiatrists confirmed that aspects of the euthanasia assessment procedure could be both favourable and unfavourable, as psychiatrists reported e.g. reduced suicide risk in some, but not all APC [18]. To date, APC’s first-hand accounts on how they experienced the euthanasia procedure are largely understudied.

Therefore, this study reports on the impact of the euthanasia (assessment) procedure as experienced by APC. We distinguish impact on three counts: 1) impact on their mental state (among others death ideation); 2) impact on their care trajectory; and 3) impact on their social relationships. This knowledge may provide clinicians and policymakers with insights to minimise negative impacts and to foster positive consequences of exploring requests for euthanasia in APC.

## Methods

### Study design and recruitment

We performed a qualitative interview study among 16 APC who had requested euthanasia in Flanders. Only Dutch-speaking APC who had made a request for euthanasia in the years (2016–2020) were included. Purposive sampling was used to ensure diversity in terms of heterogeneity in procedural outcomes (that is, diversity in terms of requests being neglected, rejected, put under review, or granted by the physicians involved, or put ‘on hold’ by the responding APC). We also ensured diversity in terms of diagnoses and age range (with a strict minimum of 18 years of age) as we expected that the impact of the euthanasia assessment procedure and the role of the social inner circle could vary according to these different patient characteristics. No further inclusion or exclusion criteria were employed.

APC were recruited via different care organisations, each of which had publicly expressed or published their own vision on how to adequately deal with APCs’ euthanasia requests; 1) the Flemish end-of-life consultation centre Vonkel, 2) the Belgian Organisation of Brothers of Charity; 3) the Flemish Association of Psychiatrists; and 4) Zorgnet-Icuro (a Flemish umbrella organisation for hospitals and care organisations).

In case the respective physician/caregiver deemed their patient eligible and able to participate, and after the physician/caregiver had given the information letter to the potential participant(s), it was up to the latter to decide (not) to contact the researchers (MV, KP, KC) by phone or mail, and were given an information letter and informed consent (see OSF).

### Interview procedure

MV or KP interviewed the APC at their location of choice for 60 to 180 min, except for one interview that was held online due to the covid-19 lockdown regulations. Interviews were audio recorded. As the interviews resulted in rich data, the authors decided to split the results over two papers. Whereas this paper focuses on the impact of the euthanasia procedure, a previous paper addressed participant’s reflections regarding the meaning of euthanasia and how it relates to suicide [21].

An interview topic list was used (see https://osf.io/j9fvz/). The following two key themes were addressed: the impact of the actual euthanasia assessment procedure and, if applicable, the impact of provisional and/or final outcomes (neglected, rejected, granted, put ‘on hold’) of the euthanasia procedure, in terms of the impact on APCs’ immediate mental state, clinical trajectory, and relationships with involved others (i.e., family, friends, caregivers). At the end of the interview the interviewer checked whether all topics had been covered. The interviews were recorded by an audio recording device and transcribed verbatim by MV. Detailed information on data management and storage can be found in OSF.

### Data analysis

As our study was explorative, i.e., not based on any theoretical framework, MV, KP and KC used an open, inductive, data-driven thematic coding procedure, consisting of four phases; 1) identification and coding of all transcripts; 2) the placing of the codes in subthemes; 3) the placing of these subthemes in overarching main themes; 4) the comparison and discussion of the findings (with all co-authors) [22, 23]. We used a model of sampling-based saturation, namely inductive thematic saturation, that relates to the emergence of new themes [24]. Data saturation was defined as 7 consecutive interviews without new themes. We recruited APC with a view to obtaining a heterogenous population in terms of socio-demographics, clinical profile, and clinical setting.

### Ethics

This research project received ethical approval from the Medical Ethics Committee of the Brussels University Hospital with reference BUN 143,201,939,499, the Medical Ethics Committee of the Ghent University Hospital with reference 2019/0456 and the Medical Ethics Committee of the Brothers of Charity with reference OG054-2019–20.

Detailed information on the measures taken to safeguard participants’ safety and wellbeing can be found in the research protocol (see OSF).

### Findings

#### Main characteristics of the participants

One APC was excluded from this study as MV and KP concluded that participant’s mental safety during and after the interview could not be guaranteed. In total, sixteen interviews were completed from August 2019 till July 2020. Participant’s main characteristics are listed in Table 2, revealing that the participants ranged in age and suffered from a variety of psychiatric diagnoses, and mostly also from psychiatric and/or somatic comorbidity.

**Table 2:** Characteristics of the study sample of adults with Psychiatric Conditions’ (APC’s) having experienced the euthanasia assessment procedure

Characteristics	*N* = 16
**Biological Sex**	
Male	3
Female	13
**Age Category**	
< 30	2
30—40 year	2
41—50 year	5
51—60 year	7
**Stage of the euthanasia procedure**^a^	
No formal advice on euthanasia obtained (yet)	9
One formal advice on euthanasia obtained	4
Euthanasia request formally granted	3
**Former/Provisional/Final outcomes of euthanasia procedures**^a, b^	
Neglected^c^	4
Rejected	4
In assessment procedure	9
No formal advices on the euthanasia request obtained	6
One formal advice on the euthanasia request obtained	3
Granted (at least two positive formal advices on the euthanasia request obtained)	3
Put on hold for a definite or indefinite period of time^d^	4
**APC’s medical condition**^a, c, f, g^	
One psychiatric disorder	4
Comorbid psychiatric disorders	6
Comorbid somatic disorders	3
Multiple psychiatric and somatic disorders	3

^a^Information retrieved from the APC during the interview, not from their medical file nor from their recruiting physician/caregiver

^b^Some APC had applied for euthanasia more than once. Seven APC reported ≥ 2 outcomes, e.g. rejected by first though accepted by the second advising physician, granted by the physicians involved but put on hold by the patient herself

^C^One APC had requested euthanasia before the law on Euthanasia came into effect. For reasons of clarity, all data, except for (the impact of) this one neglected euthanasia request were included in this study

^d^All APC cited to have “put their euthanasia request on hold for an indefinite period of time” instead of having it “withdrawn”, as mentioned in our topic list, and as literally phrased by both the interviewers

^e^Nature of psychiatric disorders according to the DSM-5 categories: Neurodevelopmental disorders (7), Depressive disorders (2), Bipolar and related disorders (3), Somatic symptom and related disorders (1), Disruptive, impulse-control, and conduct disorder (2), Trauma- and stressor-related disorders (3), Anxiety disorders (1), Eating Disorder (2) Adjustment disorder (3), Obsessive–compulsive and related disorders (1)**,** Dissociative disorders (1) and Sexual dysfunctions (1)

^f^Nature of somatic disorders: Respiratory Dysfunctions, Endocrine Diseases, Chronic/total pain, Development motor disorders, Central nervous system disorder, Visual impairment, Autosomal recessive genetic disorder and Permanent injuries after failed suicide attempts

^g^All APC (had) dealt with suicidality. Thirteen had committed serious suicide attempts

Eight participants had once had their euthanasia request neglected or explicitly rejected. At the time of the interview, nine participants had their euthanasia request under review. Three of them had already obtained at least one formal positive advice. Finally, three other participants had their request formally granted.

#### The impact on APC’s mental state, including death ideation

*“Ohhh, he really cursed and shouted and yelled at me so much that I came out weeping*”

Female, 45 years

As shown in Table 3, and regardless of whether participants were (dis)satisfied with their actual therapeutic and social relationships, a *neglect* of the euthanasia request had an adverse impact on the mental state of all participants who had experienced it. A *neglect* of the euthanasia request could entail a physician not or adversely responding to an expressed euthanasia request. They were unanimous in the view of them feeling misunderstood, their suffering not being taken seriously and not guided in finding physicians for open discussions on euthanasia. Some stated to have considered suicide again.

**Table 3:** Impact of the euthanasia procedure on Adults with Psychiatric Conditions’ state of mind, including death ideation, in the context of the euthanasia request being neglected (N), rejected (R), under review/being assessed (A), granted (G) or put on hold (P)

	MENTAL STATE	
	Favourable outcome	Unfavourable outcome
Feeling heard	Feeling recognised/heard/understood - Relief of being enrolled for future euthanasia assessment (A, P) - Being recognised/heard as regards the burden of suffering/problems in life (A, G) - Being seen as a whole (not only sick) person (G, P) Immediate impact at having request granted (G) - “euphoria”, “intense happiness”, “contentment”	Not feeling recognised/heard/understood - Being fended off (N, R) - Not being taken seriously/heard (N) - Being misunderstood as regards the burden of (invisible) suffering/problems in life (N, R)
Fear for adverse events	Less fearful of unwanted events (A, G) - No (more) fear for involuntary admissions to a psychiatric ward - Less burdened with ‘self-destructive ideation and behaviors’ - Increased ability/willingness to suppress suicidality Relief for loved ones when no formal advice on the request has been obtained - not to have burdened loved ones (N, R, A) - not to have burdened one self with further discussions on the subject (N, R)	More fears/thoughts regarding death and dying - Fearful of new (failed) suicide attempts (N, R, A) - Ambiguity about dying (fear of dying, afterlife) (A) - Time-consuming ruminations regarding *(unregulated) suicide (N, R, A) vs *euthanasia (A) - Time-consuming practical preparations for euthanasia (A,G) Distress about consequences of having request granted - stigma/labelling if APC does meet the legal criteria (A) eg. jeopardise potential opportunities in life - ambiguity about dying (fear of dying, afterlife) (A) - Uncertainty < probability of the window of opportunity narrowing/closing: (A,G) *professional backing out * legislation change *validity period of obtained positive advices (eg. physician’s retirement)
Creating Perspective, empathy	Better understanding of/empathy toward others’ perspectives *Towards physicians - Understanding/empathy towards rejection *from treating psychiatrist (R, A) *from performing physician (A, G, P) - Understanding/empathy towards physicians as regards the difficulties faced and the necessity of building sufficient reflection time (A, G, P) - Understanding/empathy towards physicians entrusted with euthanasia assessment (A, G, P) *Towards the social inner circle - Understanding/empathy: regained ability to take important others’ perspective into account (A) - Regained ability to deal with different perspectives and reactions (A, G, P)	
Perceived control	Ability to plan a good death - eg. planning and exchanging goodbyes, memorial celebration (G) - Reframing the death wish (A, G, P) eg. ‘euthanasia’ as potential safety net > < acute death request	Feelings of powerlessness, having no control (A, G) - Burden of pleading tribunal hearings’ (A, G) (pleas instead of requests for euthanasia) - Perception of being given the runaround (A, G) - Experiences of broken promises/physicians getting cold feet (A) - Distress about the uncertainty of the outcome (A) *the probability of broken promises, tightening of the law (A) (Di)stress when the outcome turns out negative - despair, hopelessness (N, R) - indignation (R) - Feeling left in the dark/to their fate to find new physicians (N) Burden of the quest in finding physicians open to euthanasia (N, R)
Fairness		Feelings of injustice, unfairness (A, G, P) - Unprofessional behaviour of physicians involved *violation of medical secrecy/confidentiality * poor communication skills (induced false hope, lack/little transparent communication between physicians involved) - Inequality of the euthanasia procedure and outcomes associated with *patient characteristics ie. the highly intelligent, verbally skilled APC and those who have important other’s approval are in the advantage * the absence of one single standard protocol approach ie. law versus a variety of guidelines
Emotional drain		Procedure itself is emotionally draining - Reluctance/burden of (repeated) self-disclosures (A, G, P) - Assessment procedure is hard/too time-consuming/over-burdening (while being exhausted) (A, G) - Being the victim of dissensions between EOL centres/played out by the dissensions between strong opponents and proponents (A)
Distress about loved ones	Relief not (yet) to burden loved ones (N, R)	Distress about consequences of the euthanasia procedure on loved ones - Burdening loved ones (A,G) - Concerns about bottled up emotions inside loved ones (N, R, A, G, P)

When asked about participants’ accounts of the *rejection* of their euthanasia request by their own treating physician or opposition from an advising physician, all participants echoed immediate feelings of emotional disturbance, anger and indignation similar to a neglect. Unwillingness of their own treating psychiatrist or physician to perform the euthanasia itself was received with more immediate understanding. Due to e.g., the often longer clinical patient-physician history and consequently, a closer emotional physician–patient connection, it was perceived more difficult for their own physician to be burdened with the task to effectively letting their own patient die a hastened death.

*“I do understand my general physician, I do understand her [refusal] because she knows me for over 20 years, and she… It's not that she's like… she has got my advance directive form and so on, and she does understand it. She just says, ‘I'm having a hard time with it.' I do understand that, hey, I do understand that. At the end of the day, I do understand that. I must be honest: I am the one with a death wish, but I wouldn't like to do that either, giving a lethal injection to someone.”*

Female, 60 years

Some participants reported that the *neglect or rejection* of their request gave them the advantage that they did not need to notify their loved ones on having requested euthanasia, so the latter would not be burdened by this knowledge. In case loved ones had been informed, two divergent discourses emerged. Whilst participants reported some relief in no longer having to discuss the subject or to burden the loved one with it, others struggled with loved ones’ attempts to discourage them to persist in their request for euthanasia and quest for new physicians. participants expressed concerns about loved ones bottling up their own emotions for participant’s sake.

Irrespective of the status of their euthanasia request – *under review, refused or granted* –participants reported ambivalent feelings throughout. The ambivalence for participants who had their euthanasia *request under review* was based on the one hand on their ability to talk openly about their death wish without fear for involuntary (re-)admission to a psychiatric ward. They felt recognized in their suffering experiences and problems. If *the first positive advice* had been obtained, participants reported being in a state of “contentment”, “intense happiness” or even “euphoria”. On the other hand, they also reported distress, mainly due to the many uncertainties, including the probability of rejection. Some participants with a *request under review* also struggled with the probability of a granted euthanasia request and its consequences for treatment options, as the ‘official’ label of having an irremediable psychiatric condition could potentially compromise their chances on treatment, social relations, and societal rehabilitation.

Similar findings emerged regarding participant’s death ideation: whilst some reported decreased suicidality, others continued to consider suicide as a plan B, e.g., if the euthanasia procedure would be too burdensome and/or time-consuming, or for some participants even as plan A, due to e.g., their growing disbelief of euthanasia as a dignified and self-chosen way of dying. Only participants who had their euthanasia request granted or on hold, reported feeling less burdened with ‘self-destructive ideation and behaviours’’, which they attributed to the feeling of being recognized in all their struggles in life and treated as a whole person (and thus not only as mentally-ill, merely struggling with death ideation as a symptom of their psychopathology, but as a person who can express a well-reasoned death ideation, resulting from e.g., being stuck in many more life domains than only the pathological one).

“Maybe that's the kind of strength I have now, like: 'No, I'm not going to take overdoses, I'm not going to be self-destructive, I'm going to hold back on myself. And maybe that's my strength, despite everything I've been through, I think. Yes, it must be something like that. And that strength came through [names two new health care professionals]. I may have had it already in me, but they were able to strengthen it. And I am so grateful to them. No matter how difficult it is. I mean, that is so precious. So, it's not that obvious at all. It is not obvious at all. If I had met those two people earlier, my life would have been different. I would probably have had a different life, cause maybe I wasn't ready either, I don't know. I don't know. But they have come on my path and things have changed and... I feel so understood and respected. Not just sick and incompetent. Not just a label, not me with a label, not just a label.

Female, 55 years

Ambivalence was also found in participants with a *granted* request. They literally phrased that they were in an immediate state of e.g., “intense happiness”, “being blessed” and/or “intense relief”. However, after some time, ambivalence re-appeared. This may be understood as no longer having to ponder on how to die (by means of suicide or euthanasia) which on the one hand allows more time to try out alternatives to death (i.e., euthanasia as a safety net). On the other hand, participants also have to deal with preparations for euthanasia (e.g., when, where and with whom) and many perceived uncertainties such as the validity period of obtained positive advices (in case of physician’s retirement or a legislation change).

Some participants mentioned more peace of mind to suppress suicidality and hence more time to take all aspects of dying or alternatives to death into account. The latter result is explained as follows: the benefit of following a two-track approach during the whole procedure, in which not only participant’s eligibility for euthanasia had been assessed, but also alternatives to death, including rehabilitation, always in dialogue with the participant. Others remained torn between their wish to die versus the burden of leaving behind bereaved relatives. However, some other concerns remained, like finding a performing physician or, when found, the likelihood of her change of mind, retirement or passing away. In addition, uncertainty was experienced regarding the validity term of the advice obtained.

#### Impact on death ideation

*“Yes, it is a very long procedure. You must be very patient and when do you apply for it? At a time when you really feel exhausted, then you ask for it. And then they expect you to go everywhere, to have all those conversations, but you don't have that energy anymore, they… But that is what they expect. I think, someone who has a physical problem and already receives palliative care, they're not going to say: "Okay, you'll get euthanasia, but first you have to run a marathon, huh, so you better start going to the physiotherapist." They don't do that, but we are expected to do so, we still have to be able to do everything, that's deemed normal.”*

*Female, 50 years*

All participants applied for euthanasia on the assumption that they would have some control over their own process of dying, leading to a dignified death. However, most participants, even those with a granted euthanasia request, reported that during the long and exhausting assessment procedure, they had come to see the idea of a *self-chosen death* as an illusion, considering euthanasia as a medical favour that they had to plead for. They further tackled the following as undermining the assumption of euthanasia as ‘dignified death’: 1) poor communication between the physicians involved and towards the patient (from physicians and caregivers inducing false hope as regards the duration and outcome of the procedure, to the violation of confidentiality), 2) the inequality of assessment procedures within and between different institutions, and 3) the uncertainty about the outcome of a recent euthanasia case being subject to criminal investigation (e.g. possibly leading to future changes in physician’s attitudes, to broken promises or to future changes in legislation at the participant’s expense).

Most participants held that the euthanasia procedure is too time-consuming and overburdening, due to e.g., the many self-disclosures that had to be expressed repeatedly to at least three physicians during what some literally phrased as ‘pleading at the tribunal hearing’. Finally, they criticized the perceived unequal assessment favouring highly intelligent, verbally proficient participants, and participants with less complex clinical pictures—even if they themselves were the ones being advantaged. Also, although the procedure was experienced as highly burdensome, some did not seek the easy way to get their wish fulfilled, as some participants were willing to have at least two formal *positive* advices (instead of the 2 legally required advices of which the nature – positive or negative – is not binding), for the performing physician’s sake.

*“I had, in good conscience, decided together with my general physician, who wanted to be the performing physician and still wants to be, that we had to obtain POSITIVE advices. Because, and I think that this is very important and something that a lot of people still underestimate… My... The fellow peers that I have known and still know, uh, we are really concerned about the welfare of the physician who is going to help us, aren't we? Because I hear that a lot in the media and each time again, it hurts me a lot, that one is saying like, ‘yes, but well, those POOR physicians who...’. First of all, they are not compelled to do it. If my GP had said 'no, I don't want to do it'. I always told her that. If you don’t want to, then just say so. We have been very transparent about that, from the beginning. And that's the only way to handle it, I think. You have to be very honest with each other and ask, 'Are you ready for this'? "Yes, okay then, but take some time to think it through, and if you don’t want to, I'll seek someone else, right?”*

Female, 33 years

#### The impact on participant’s clinical trajectory

*"With Dr [name attending physician], I can have these conversations about euthanasia about five times a year. And just that, just knowing that I could discuss it with him every time, without getting a stigma, huh? Knowing that it might be possible one day, might even be a manner that enables you to continue to live, eh? That you are being taken seriously, that you are indeed allowed to talk about it and that, because of that, you don't get a certain label of "What a strange patient is this? Do you really have to put me through this? Does she really have to burden me with this? It's not what a physician is meant to do.” So that you have a safe setting somewhere where you can go to and have it discussed, and when you leave, that you can also step back into your life. And I realise that this is very strange and difficult to understand, even if you were to tell people about this, because on the one hand, you are on a heavy therapeutic trajectory, in which you put every focus on life, and in which you make all kinds of plans for the future and advancing your future, but then, on a parallel track, you are on a trajectory in which it is possible that I might take that turn towards euthanasia. So, I am actually following a two-track trajectory."*

Female, 43 years

As shown in Table 4, all participants whose request had been *neglected* (e.g. the request falling on deaf ears) phrased that it had damaged the actual therapeutic relationship, resulting in treatment noncompliance, and eventually in quitting the current therapy. participants who felt dismissed by their physician reported an irreparable mistrust in their physician’s professionalism and immediately quit their current therapy.

**Table 4:** Impact of the euthanasia procedure on APCs’ clinical trajectory, in the context of their euthanasia request being neglected (N), rejected (R), assessed (A), granted (G) and put ‘on hold’ (P)

IMPACT ON THE CLINICAL TRAJECTORY	
Favourable	Unfavourable
Continuity of care (R, A, G)	No continuity of care (R) - Treatment abandonment by the patient (N) - Treatment abandonment by the caregiver (R)
Open discussion about the death track within treatment trajectory - discussion of death ideation and euthanasia encapsuled in therapy (with respect, honesty and integrity) (R, A, G) - Being able to openly express the request and have it assessed (A, P) - Serene/caring talks about death (A, G, P) - Dialogic, compassionate approaches (A, G, P)	No discussion of the death track within treatment trajectory - talks on death ideation/euthanasia not being encapsuled in the existing treatment trajectory (R, A)
New referrals & treatment approaches - Meaningful referral (R, A, G, P) *to new/additional treating physicians *to additional caregivers - Meaningful advices/suggestions (e.g. new diagnosis, reframing death ideation and other problems in life) - preparedness to continue treatment (R, A, G, P) - preparedness to halt acquired treatment resentments (G) - Encouraged/empowered to undergo further/additional diagnostic testing/ treatment options (A, G, P)	Referral & further treatment burden - no meaningful referral (R, A) - Burden of additional psychodiagnostics testing/therapy (A, G) Poor patient-commitment, just undergoing additional testing/treatment to get file approved/hiding behind irrelevant diagnoses/events/occupational therapy (A)
	Souring patient—physician relationship during the euthanasia trajectory - Directive approaches of physicians involved (A, G) - Breakdown in relationship with treating physician (e.g. when verbally attacked by the physician, being disinformed, useless referral) (R, A) - Mistrust in physicians involved (A) (cf. instrumental burden + in case of violation of confidentiality)

In case participants’ treating physician *rejected* active engagement in the euthanasia request, the impact this could have on participants’ clinical trajectory varied. Some participants reported being verbally attacked, wrongly informed on the legal aspects of euthanasia, or deliberately misled by their physician (e.g., suspicion that the physician had referred to another physician, knowing that the latter would also reject participant’s euthanasia request). Irreparable mistrust, and discontinued therapy was also cited. Other participants mentioned no changes in treatment adherence once their primary emotions and disturbing feelings were processed and the reasons for rejection were thoroughly discussed with the treating physician. These participants also appreciated their physician for being open to hold serene talks about death ideation and euthanasia in forthcoming therapeutic sessions and for meaningful referral (i.e., referral to another physician, willing to be actively engaged in the euthanasia procedure and holding an open stance towards euthanasia).

Treatment adherence was reported by all participants who had their request in review, granted or put on hold, although ambivalence was noted throughout the euthanasia assessment trajectory.

As for the subgroup of participants who put their request on hold, some participants dealing with ambiguous feelings and thoughts about the meaning of life and death, reported to have found reassurance in the fact that their euthanasia request and medical file had been registered, handled, and cared for by (at least one) ‘competent and trustworthy’ psychiatrist. These and one other participants who had not yet obtained a formal advice on their request felt sufficiently reassured and empowered to explore new paths of rehabilitation, knowing that they could explore the death track more actively in case their personal situation would deteriorate, and their death request would become more enduring and consistent. One participant who had already obtained (more than) the two required legal advices, only felt empowered to explore new ways of living once the euthanasia request had been granted with the physicians’ reassurance that it remained an option to fall back on.

*"The right not to be forced to be here, but to be allowed to be here, is what made me stay here. It ensured me that I could be here, that I could continue to live here. That's really how it was for me, the right not to be obliged to live here made it possible for me to live. And I have put the procedure on hold now, but still, I know it is not far out of reach and knowing that still helps me. It is not out of reach and knowing that helps me out in the most difficult moments."*

*Female, 47 years*

The impact of the euthanasia procedure on participants’ treatment is illustrated by following experienced changes to their clinical trajectory:

1) adequate help and treatment for a new diagnosis, 2) a transition from a rather restrictive to a more patient-centred care model with a focus on rehabilitation, in which self-destructive behaviours could be reframed when identifying remaining functional potentials, and 3) with additional support for not only medical but all problems faced in life (e.g., autism coaches helping them with administrative issues).

However, whilst some participants felt empowered to give alternatives to death a fair chance of success, others perceived the suggestions on additional psychodiagnostics, additional treatment, and other rehabilitation options as over-burdening and futile. These participants consented to proposed additional treatments only with a view to obtain approval for euthanasia.

#### The impact on participant’s social life

*“And euthanasia, well, you can say goodbye, the people who will stay here can be prepared for that moment. I would do anything to achieve that. I wanted them to meet with my psychologist. I wanted them to have guidance during the procedure, yes. Saying goodbye also, I, I knew who I’d allow to stand at my bedside, yes, I found that, I found that so much more serene than just leaving by surprise.”*

Female, 47 years

As shown in Table 5, whereas some participants took the initiative to inform their loved ones about their euthanasia request and procedure, or to involve them to a certain extent, other participants were urged to do so by their physicians. For some participants, somehow involving their relatives was unjust toward themselves (e.g., for fear of violating medical confidentiality or that strong opposition would compromise their chances of euthanasia), unnecessary (especially in the case of a tentative euthanasia request), or even undesirable (because of the emotional burden and responsibility it places on intimates as well as the possible conflicts it provokes between relatives who were and were not informed or more deeply involved).

**Table 5:** Impact of the euthanasia procedure on APC’s social life, also in the context of their euthanasia request being neglected (N), rejected (R), assessed (A), granted (G) and put ‘on hold’ (P)

IMPACT ON SOCIAL LIFE	
Favourable	Unfavourable
Receiving understanding & more emotional support - Increased attention, compassion (R, A) - More serene talks about death (A, G) (with respect, honesty and integrity) - Opportunity to share the emotional experience (A, G) - Received blessing (A, G) - Additional support/understanding from ‘similar’ peers (eg. from experts by experience) (A, G) - Ability to learn from ‘similar’ peers (eg. joined forces to make life more bearable/to see alternative options) (A, G)	Not being supported or understood - No/little understanding for APC’s perspective (A, G) - Adverse attempts to change APC’s mind (R, A) - Negative reactions/conflicts (R, A, G) - Non-committal approaches/reactions (R, A) - No mutual understanding due to conceptual confusion (legal terminology) (A)
Rebuilding social relationships - Opportunity for rehabilitation of existing social relationships (deeper connection) (A, G) - Empowered to open-up/build new relationships (G, P)	Crumbling relationships - Resignation from family and other ‘social obligations’/ further erosion of the network (R, A) - Decreased sense of belongingness (R, A) - Increased feeling of being ‘alienated’ (R, A)
Receiving more practical support - Offering eg. transport and shelter after consultations with physicians (A) - Suggesting potential helpful/comforting books/movies (A)	
Support for important others possible - Opportunity for loved ones to receive support (A)	
	Difficulties with involving and managing interactions with important others - No/little advice/guidance on how to inform the inner circle - Informing relatives is deemed unfair (A) *wrong as it is only a measure to protect physicians from deontological/ juridical complaints *unjust to exclude (eligible) APC from euthanasia if someone/some members would strongly oppose to it *it puts a heavy burden on the few one’s involved *it may provoke conflicts/ruptures after APC’s death - Reluctance to hurt loved ones needlessly (eg. when informed in an early stage) (R, A) - Incompatible objectives patient versus relatives or among relatives (A) - Practical difficulties of informing the inner circle (i.e. how, when and where to inform whom) (A) - Emotional difficulties: • to cope with mixed reactions/stages of grief (A, G) • when reactions within the social circle (A, G) • fear of/difficulties to cope with meddlers outside the close inner circle (A)
	Comparing own situation with fellow peers (mirror-window) - Concerns regarding fellow APC making precarious use of the euthanasia procedure (A, P) - Difficulties to cope with the loss of fellow peers in inpatient settings (suicide and euthanasia), especially in case of omerta rulegiving (P) ie. APC were ‘forbidden' to talk to fellow peers about their own or another fellow peer’s euthanasia request/euthanasia procedure/attempted suicide/suicide)

In case loved ones had been informed or involved during the euthanasia procedure, divergent reactions emerged. Some participants experienced informing or even involving loved ones as positive, provided there was a serene atmosphere during the euthanasia procedure, based on reciprocal understanding and empathy. These participants valued the opportunity to share this emotionally difficult trajectory with loved ones and of supporting each other through the euthanasia procedure. As mentioned earlier, it provided them reassurance that their loved ones do not have to keep their feelings bottled up. Rehabilitation of existing troubled social relationships or broken relationships was reported by some but not all participants with euthanasia requests rejected or in review. The opposite also occurred and resulted in a decreased sense of belonging or an increased feeling of being ‘alienated’. Some participants reported new relationships with understanding and supportive peers, from whom they could learn how to make life more bearable.

The reasons why there was reticence to inform or involve the inner circle were the following: 1) the lack of tools and support for participants to engage in the conversation with their loved ones (i.e. how, when and where to inform whom), 2) the anxiety to hurt loved ones, and 3) the burden to have to cope with the inner circle’s emotional reactions, whether absent, negative, mixed, or with disagreements, and 4) concerns on having to deal with potential meddlers from outside the close inner circle.

The following experienced disadvantages were reported in the relationships with other participants: 1) concerns regarding peers making precarious use of the euthanasia procedure, e.g., the perception of euthanasia used as a cry for attention, 2) the difficulties to cope with omerta rules in inpatient settings, i.e., when participants were ‘forbidden' to discuss a euthanasia request or procedure with peers, and 3) to cope with the loss of fellow peers, be it by means of suicide or euthanasia.

## Discussion

This qualitative study revealed a multifaceted impact of the euthanasia assessment procedure on APC. Whereas thoughts of suicide remain present to a certain extent, being in the assessment procedure allows some APC to reconsider alternatives towards life, and also to attempt new treatment options. However, many APC experience ambivalence about the supposedly inherent desirability and dignity in euthanasia. Worries also surfaced about the effects of involvement of APCs social circle, and about the impact it could have on them.

### Strengths and limitations

To the best of our knowledge, this is the first study that emphasised the impact of euthanasia procedures on APC. As previous studies among APC focused on the reasons why APC request euthanasia [19, 21, 25, 26], this study systematically investigated the impact on their mental state, current care trajectory and social relationships.

Another strength of this study is the minimal risk of social desirability answers, as the interviewers were not involved in the APC’s euthanasia procedure, full confidentiality was guaranteed and the APC were not pressured to phrase their views and experiences in any direction, which resulted in very rich, unique and detailed data. Moreover, the sample can be considered heterogeneous in terms of clinical diagnoses, age ranges, different stages in the euthanasia procedure and the APC being recruited via multiple mental healthcare institutions and organisations, which maximised sample completeness. Thematic data saturation had been reached as no new themes emerged after the 7^th^ interview.

A limitation of this study is the potential lack of thematic saturation *per outcome* of the euthanasia assessment procedure. Furthermore, selection bias may have occurred as: 1) the sample was recruited by the APC’s physicians; and 2) the euthanasia procedure of most of the APC interviewed was affected by recent and potential changes in euthanasia practice, due to the recently published deontological code recommending more strict due care criteria [17] (e.g. obtaining the formal advice from at least 2 instead of 1 psychiatrists) and the legal and emotional consequences regarding one high-profile euthanasia case being brought to court. Given these cited limitations, external validity of the findings may have been limited.

Finally, our findings cannot readily be generalized to other countries that allow medical assistance in dying for adults with psychiatric conditions, as in each country, the specific mental health care context, the stipulation of legal due care criteria and its implementation in the clinical practice, and guidelines differ to a certain extent.

However, the findings of our study are important for all jurisdictions to take into account, as it is the first study that makes use of patient’s first-hand accounts and therefore, shed some new lights on the practical-clinical as well as medical ethical and even legal debate on how to adequately handle these trajectories.

### Interpretation of main findings

APC clearly benefited from being listened to, being recognised in their suffering and valued as a person, and having their euthanasia request being taken seriously. This finding supports the so-called ‘therapeutic effect’ of euthanasia assessment procedures as it may suppress suicidality [26, 27] and may even offer sufficient peace of mind to give alternatives to death a fair chance of success once their request is positively advised or granted [26, 27]. However, this does not apply in all APC and if so, it only seems to have an ephemeral effect, as most APC continued to struggle with ambivalence, irrespective of the (provisional or final) outcome of their euthanasia assessment procedure. The ambivalence was present on three counts: ambivalence toward 1) longing for death, 2) toward euthanasia as a desirable alternative to suicide, and 3) toward euthanasia as a dignified way of dying.

First, ambivalence toward death can be partially explained by different motives for requesting euthanasia. A previous study, also based on interviews with this sample of APC, revealed that whilst some APC make an active euthanasia request, others request it in a more tentative, exploratory or prospective way [21]. Those euthanasia requests may be considered a cry *of* unbearable pain and suffering instead of a ‘cry for help to exit life’, as these APC seem to seek the physician’s help to recognize and to alleviate their burden of suffering. The ambivalent feelings surrounding a potentially granted request (that may compromise their chances on treatment, social relations, and societal rehabilitation) must also be interpreted as such. Note that requesting euthanasia was not simply a recourse of realizing death for all APC, as some request euthanasia to hear of one’s ineligibility for it, to have their hope restored in terms of finding more ways to make their life more bearable, if not clinically, then psychosocially or existentially [21].

Second, ambivalent feelings toward euthanasia and suicide suggest that APC view both as means to the same end, with euthanasia being more dignified and preferable than suicide but very difficult to obtain. Building on this, our findings suggest a growing realization that they are in control of neither the euthanasia procedure nor the outcome, which leads them to doubt whether euthanasia is a dignified way of dying for them. Almost all interviewed APC, even those who had their request granted, experienced the whole euthanasia trajectory as an emotional tug-of-war, due to the many self-disclosures and ‘pleads’, to (the difficulties to deal with) outcome uncertainty, and the presumption of unequal assessment procedures. The latter may point to a tension between the physician’s autonomy to opt for a strict adherence to the legal conditions or the implementation of additional due care criteria on the one hand, and the burden of this non-uniform procedures on the APC.

Another main finding is that the treating physician’s rejection of the euthanasia request does not necessarily compromise the therapeutic treatment, provided: 1) good physician–patient communication in which the reasons behind the rejection are well-motivated, 2) meaningful referral, 3) openness to discuss the (ambivalence toward the) death ideation and the euthanasia procedure in upcoming therapeutic sessions. In contrast, neglecting the euthanasia request seems to have only unfavourable consequences. This finding suggests that both psychiatrists and APC may benefit from open and serene discussions about death and euthanasia. The scenario of losing a patient to either another therapist or to death seems more likely to happen if the euthanasia request goes unheard or faces a wall of impenetrable incomprehension.

As regards the impact of the euthanasia procedure on APC’s social relationships, divergent discourses emerged. While some APC reported valuing some relatives being involved in the euthanasia assessment procedures, others raised concerns or strongly opposed their involvement. The stronger position and (informal) role for APC’s social inner circle is not a legal requirement, yet strongly recommended by the recently published advisory texts and effectively implemented in today’s euthanasia practice as additional due care criterion. Motives for engaging relatives are many. The triadic dialogue between the APC, their physicians and relatives may enhance the quality of the euthanasia assessment. Heteroanamnesis can be of great value for physicians as it can further elicit the family history and lead to a better understanding of the APC and their relational and situational context. In turn, relatives may gain deeper understanding on the APC’s suffering and the meaning of their euthanasia request, and their involvement may soften their mourning in case the euthanasia is performed. Of course, psychiatrists may also welcome the involvement of APCs’ relatives in order to avoid disgruntled relatives after the fact. APC themselves may find additional support from loved ones, which may lead to further social rehabilitation.

However, some APC expressed critical concerns about the feasibility and desirability of involving relatives, as e.g., practical advises on when and how to involve which relatives to what extent, and how to manage potential conflicts, are lacking. This would seem to be subject of thorough discussion between patient and physician. In addition, we are left with the question whether all relatives would be willing to be informed and engaged during the euthanasia procedure. To date, only one Dutch interview study with 14 ‘relatives’ (parents, life partners, siblings, friends and/or fellow-patients) of these specific euthanasia requestors is published and revealed the emotional impact the trajectory has on each of them, and their needs of being recognised and not completely being side-lined [28]. The study also showed mixed findings on the desirability of being involved to a greater extent – which is highly recommended in the Dutch and Belgian medical codes of conduct – in the euthanasia assessment procedure (cf. the moral dilemma on dyadic patient-physician versus triadic patient-physician-relatives autonomy) as well as on the specific needs for aftercare [28].

### Implications for further research on psychiatric practice and policy

As our study illuminates that the euthanasia assessment procedure may be beneficial or/and detrimental to APC’s mental state, suicidality, and their current care trajectory, further research should elicit the determinants of when and why the euthanasia procedure may have a therapeutic effect on some, but not on all APC. In addition, research should further explore the notion of ambivalence towards *whether* and *how* to die and how it relates to the concepts of e.g., being in want of control and dignity, and self-determination and connectedness in dying, and also to what extent this phenomenon also befalls non-APC populations requesting euthanasia. The perspectives of physicians, caregivers and APCs’ social circle should also be studied regarding this matter.

As regards practice, it is quintessential for psychiatrists to anticipate the emotional impact of euthanasia procedures on APC. As our findings confirm the needs of APC to feel listened to, being taken seriously and recognized in their suffering, it is recommended that psychiatrists embrace the two-track approach, as suggested by advisory texts [16]. This two-track approach is characterized by focusing both on the *life track* by means of e.g., continuity or reassessment of treatment, and on the *death* track by means of assessing the APC’s euthanasia request. The rationale behind this two-track approach is that it should not be ruled out that the euthanasia request is the expression of an APC not seeking help to die but to alleviate the suffering or to anticipate future suffering. For that reason, while on the *death* track the reasons for and the eligibility of APC’s euthanasia request are explored, a life track is pursued simultaneously, in which alternatives to death are explored from a medical, psychological as well as from a social and existential perspective.

As our study reveals the negative impact of a neglected euthanasia request on APC’s mental state and treatment trajectory (see Table 3), it is highly recommended that treating physicians who would rather not actively engage in the euthanasia assessment, focus on the life track and timely refer the APC to a colleague willing to further explore the APC’s request. Meaningful referral to a colleague or experienced institution, e.g. end-of-life consultation centre is legally enforceable since the revision of the Belgian Euthanasia Law in 2020 [29]. Following this 2 track approach implies additional safeguards to the legal criteria (e.g. treatment non-abandonment), as recommended by the Belgian guidelines that have been published in recent years [16].

But even if a psychiatrist is willing to actively engage in an APC’s euthanasia procedure, expectation management seems to be of utmost importance. Through clear and careful communication, it should be explained to the APC that euthanasia is not an enforceable right nor a subjective medical decision but encompasses the assessment of stringent legal criteria. A proactive approach in which the whole procedure is explained, and all potential outcomes discussed before the assessment procedure is initiated, is considered needed to prevent severe distress that compromises the treatment trajectory. For this reason, the Dutch guideline stipulates that physicians need to respond with a ‘*No, unless…*’ to death requests from APC [30].

If the request is rejected, it should also hold the engagement that in the future, there is the possibility of having a re-evaluation of the request if circumstances have changed, due to e.g., the effects of additional treatment options or other aid in life. That said, it is of utmost importance to avoid therapeutic tenacity, as inducing false hope in patients by means of e.g., not properly informing them on treatment outcomes, offering false premises, gold-plating positive and omitting negative probabilities, or ignoring treatment fatigue, can also result in an acquired loss of the ability to hope as well as in the ability to trust the physician’s judgement [31].

It is of utmost importance to explore and deal with the emotions of disappointment, anger, and despair. This should be dealt with in the parallel consultation sessions (i.e., on the life and death track) to prevent the negative impact on the ongoing treatment. Given that suicide remains on APC’s minds in some cases, it is also needed to take it seriously, but to make explicit that threatening with suicide compromises sound decision making and therefore an APC’s chances of having their request granted. Both in the condition of granting or rejecting the request the impact of spill-over in the parallel treatment sessions must be minimized.

As regards policy, the main issue that needs medical-ethical and regulatory attention and reflection, is if, when, how and to what degree to involve the relatives of the patient. It goes without saying that engaging in APC’s euthanasia procedure is emotionally and professionally very demanding, especially as the physicians involved usually want to take the needs of APC’s loved ones into account [18, 32]. Our findings also revealed ambivalence in some APC being torn between their death seeking behaviour in order to alleviate their suffering versus the reluctance to make their relatives suffer from their death seeking behaviour. However, whereas some APC value their relatives’ involvement, others reflected on the reasons not to strengthen the role and position of relatives. This is a rather complicated issue, also from a legal point of view. Some of the participants pointed to e.g., the potential violation of their legally binding patient right on medical confidentiality and privacy, and the risk that a stronger involvement may undermine their autonomy (the underpinning principle of both the Belgian Law on Patient Rights and the Law on Euthanasia).

Other issues that according to our study deserve consideration due to the impact on APC are: differences in policies between organisations; differences in assessments based on APCs’ verbal and cognitive abilities; validity periods and conditions of positive advices and granted requests.

Finally, and as mentioned in the introduction, additional (procedural) criteria for this specific patient group are formulated in the recently published guidelines. Participants already had the strong idea of having to jump through a larger number of hoops than other patient groups, as these euthanasia trajectories often entail e.g., more recurrent consultations with more physicians, and a stronger involvement of other health care personnel and relatives. Although there are reasons for adding procedural due care criteria for the non-terminally ill due to e.g., their presumed higher life expectancy, in order not to produce a discriminatory effect or even a violation of their patient rights, a thorough juridical and medical-ethical debate on this matter, and how this should be properly implemented in practice is highly recommended. This is also an important lesson for other jurisdictions which have or consider similar medical assistance in dying procedures, e.g., Canada, as in most of these jurisdictions, family members’ involvement in health-related decisions has become a cultural norm but is not legally required due to the underpinning principle of upholding medical confidentiality and patient autonomy.

## Supplementary Information

**Additional file 1:** **BOX 2.** List of Coded Fragments per Table in English.

## Data Availability

Although data property rights are owned by the Vrije Universiteit Brussel (VUB) and Ghent University, researchers KC, KP and MV held intellectual rights on data storage and use, only to the extent necessary for the abovementioned scientific research purposes, until the study was approved for publication. However, as for transparency and reproducibility, i.e., good scientific practice, full (though anonymized) data can be made accessible following procedures from all 3 Medical Ethics Committees involved. To access the supplementary materials, see the Open Science Framework repository at https://osf.io/xq7mz/.

## Abbreviation

APC: Adults with psychiatric conditions
