# Supplementary material for: The impact of the euthanasia assessment procedure: a qualitative interview study among adults with psychiatric conditions
Source: BMC Psychiatry. 2022 Jun 27;22:435. doi: 10.1186/s12888-022-04039-2 (PMC9235145; doi:10.1186/s12888-022-04039-2)
Supplement: Supplementary file 1 — Additional file 1: BOX 2. List of Coded Fragments per Table in English. [file 12888_2022_4039_MOESM1_ESM.pdf]

BOX 2: List of Coded Fragments per Table in English

Table 2: Impact of the euthanasia procedure on Adults with Psychiatric Conditions' Mental State, including on their death ideation

	MENTAL STATE	
	Favourable	Unfavourable
PROCEDURE-related		
Feeling heard	Feeling recognised/heard/understood<br>***"Finally, I felt heard, I felt understood, I felt supported, I felt I was being carried."***<br><br>Female, 51 years	Being fended off<br>"***Ohhh, he really cursed and shouted and yelled at me so much that I came out weeping***"<br><br>Female, 45 years
Fear for adverse events	Less fearful of unwanted events<br><br>***"But on the other hand, I do feel that I am stronger than I was a while ago. Do you know why? I'm here with a huge amount of medication lying around, and it hasn't occurred to me to take an overdose and that's a huge difference. Before, in an emotional crisis, I used to take a whole box of anything and everything until I was lying here in a coma and the ambulance had to come to pick me up, and things like that. And now I can leave all that behind. I'm not going down that road anymore. So now I'm like, 'I'm going for survival, and I've got to try and stay strong now and not give in to self-mutilation and self-destructive behavior.' Even though I'm so used to that and it's so easy to give in to that because it became so normal... that threshold has been so lowered... Yes, well and now I have that... Now I have like some sort of power within me to say no to that. I'm not going to do that anymore, I'm not going to start with impulsively swallowing pills, taking the whole box of meds and ending up in intensive care. That is my strength, at this moment."***<br><br>Female, 55 years	Fear of actually dying, afterlife (Ambiguity about dying)<br><br>***"Ah yes, what I did notice when I applied for euthanasia, and these are two things I have never read about in the euthanasia debate... First, I have a very strong fear of death, I must overcome something terrible in me for that, and I have never really succeeded to do so; so as soon as I asked the question 'I want to die, give me euthanasia', I was at the same time afraid of 'what if he immediately approves and goes along with it? That is because I am so afraid of death. And secondly, what I also never read about: it is an unknown fact what would happen after death and who knows, the afterlife may be even worse. No one has ever returned from death. It is assumed that the suffering stops then, but no one, no one has ever returned from it. Who says it won't get worse or that there will be another form of life after this, life that nobody knows about? I am agnostic, so I don't know what might come afterwards. And those two fears also relate to one another. My fear of death relates to me not knowing what may come after death. And then, I am actually taking a gamble, aren't I? That is how I feel. The gamble is that I hope that the suffering would stop after death, but that is not a certainty, is it? And I've never read anything about that in the euthanasia debate, even though I think it's a logical question, don't you?***<br><br>Male, 54 years<br><br>AND<br><br>Distress about consequences of having request granted<br><br>***"I do see an impediment, don't you? At this moment, once you obtain the approval for euthanasia, the approval is valid for an indefinite period of time. As I understand it, the attending physician will always seek confirmation, will assess if the euthanasia request is still justified, but shouldn't this approval be limited in time?***

		The one person I just spoke about, the one who received the approval so quickly and this for more than five or seven years now, well, this person is still alive, he also wants to continue living, but he also feels the hindrance of the obtained approvals, because it renders many things impossible in his actual life. *(Interviewer: Yes? Even nowadays? So you can still see the impact...)* ***Yes, I talked to him about the idea of a romantic relationship, and he said to me 'well, that's no longer an option for me, nobody wants me anymore'. The people that I know, like [name of another fellow peer], yes well, assume that she would meet a nice man and fancies a relationship with him, then that man would be troubled by the idea 'I'm having a relationship with someone who suffers unbearable and irremediably. Personally, as a partner, I think I would not be able to bear that either, with my partner. So, I think to myself, she is making it difficult for herself to live. (...) You'll block off a lot of things for yourself by already having obtained that approval, yes.***<br><br>Male, 54 years
Perspective taking/empathy	Better understanding of/empathy toward others' perspectives<br>*As regards physicians<br>***"I had, in good conscience, decided together with my general physician, who wanted to be the performing physician and still wants to be, that we had to obtain POSITIVE advices. Because, and I think that this is very important and something that a lot of people still underestimate… My... The fellow peers that I have known and still know, uh, we are really concerned about the welfare of the physician who is going to help us, aren't we? Because I hear that a lot in the media and each time again, it hurts me a lot, that one is saying like, 'yes, but well, those POOR physicians who...'. First of all, they are not compelled to do it. If my GP had said 'no, I don't want to do it'. I always told her that. If you don't want to, then just say so. We have been very transparent about that, from the beginning. And that's the only way to handle it, I think. You have to be very honest with each other and ask 'Are you ready for this'? "Yes, okay then, but take some time to think it through, and if you don't want to, I'll seek someone else, right?"***<br><br>Female, 33 years<br><br><br>*As regards the social inner circle<br>***"So that weight fell off my shoulder and was replaced by so much inner space. Suddenly I had the opportunity to empathise with, for example, what my mother felt, what my sister might feel, what my brother might feel, what my neighbour might feel, yes, and my friends and acquaintances. Suddenly, there was enough space to reflect about this and that was a great relief, that I had received the necessary recognition from [name of 1st***	

	advising physician who gave the positive advice]. I got that relief and recognition from her, and she gave me the gift of also being able to give recognition to others. I did not have that space before. On some practical level I could still see that, uh, my mother was going to lose one of her children, so from a practical perspective, I could see that, but I could not empathise with it, emotionally I could not... I didn't have that space. I didn't have that space to evoke that emotion, or create it, or to get it or feel it or whatever."<br><br>Female, 51 years	
Perceived control	Reframing the death wish<br>e.g. 'euthanasia' as potential safety net and not an acute death request<br><br>***"The right not to be forced to be here, but to be allowed to be here, is what made me stay here. It ensured me that I could be here, that I could continue to live here. That's really how it was for me, the right not to be obliged to live here made it possible for me to live. And I have put the procedure on hold now, but still, I know it is not far out of reach and knowing that still helps me. It is not out of reach and knowing that helps me out in the most difficult moments."***<br><br>*Female, 47 years*	Feelings of powerlessness, having no control (A, G)<br><br>***"No, (the procedure) is beyond your control. You have to comply with anything and everything."***<br><br>*Female, 43 years*
Fairness		Feelings of injustice, unfairness (A, G, P)<br>***"Yes, all these conversations with all these physicians. I don't call these 'conversations' anymore. I call them: going to plead your case, going to argue your case. I think, if you can uphold it well, that is, if you can explain it well, that you will get it. And if you have the right people around you, you will get it. And if you don't have the right people around you, you won't get it."***<br><br>*Female, 45 years*
Emotional drain		Procedure itself is emotionally draining<br>***"Yes, it is a very long procedure. You must be very patient and when do you apply for it? At a time when you really feel exhausted, then you ask for it. And then they expect you to go everywhere, to have all those conversations, but you don't have that energy anymore, they… But that is what they expect. I think, someone who has a physical problem and already receives palliative care, they're not going to say: "Okay, you'll get euthanasia, but first you have to run a marathon, huh, so you better start going to the physiotherapist." They don't do that, but we are expected to do so, we still have to be able to do everything, that's deemed normal."***<br><br>*Female, 50 years*
Distress about loved ones		Distress about consequences of the euthanasia procedure on loved ones<br>***"Because I felt that [name of the life partner] was keeping a lot to himself and that hurt me. I didn't want him to carry on like that. I wanted him to be able to***

		express his feelings and thoughts and... to openly speak about it and... But that was difficult for him."”<br><br>Female, 55 years

Table 3 Impact of the euthanasia procedure on APCs' clinical trajectory

THE CLINICAL TRAJECTORY	
Favourable	Unfavourable
Continuity of care<br><br>***"With Dr [name attending physician], I can have these conversations about euthanasia about five times a year. And just that, just knowing that I could discuss it with him every time, without getting a stigma, huh? Knowing that it might be possible one day, might even be a manner that enables you to continue to live, eh? That you are being taken seriously, that you are indeed allowed to talk about it and that, because of that, you don't get a certain label of "What a strange patient is this? Do you really have to put me through this? Does she really have to burden me with this? It's not what a physician is meant to do." So that you have a safe setting somewhere where you can go to and have it discussed, and when you leave, that you can also step back into your life. And I realise that this is very strange and difficult to understand, even if you were to tell people about this, because on the one hand, you are on a heavy therapeutic trajectory, in which you put every focus on life, and in which you make all kinds of plans for the future and advancing your future, but then, on a parallel track, you are on a trajectory in which it is possible that I might take that turn towards euthanasia. So, I am actually following a two-track trajectory."***<br><br>*Female B, 43 years*	No continuity of care (R)<br><br>***"Yes, first I followed day treatment twice and after that, I went to a revalidation centre for psychosocial revalidation purposes. And uhm, yes, well, because I talked about euthanasia, I wasn't allowed to stay there anymore, because normally, well, they need to get permission from the RIZIV [Belgian National Institute for Health and Disability Insurance]. And normally, depending on your diagnosis, you can stay there for a year and a half to two years. And I had already been there for one year, but they didn't want to make a new application for me because they thought that applying for euthanasia would mean to be ruled out for rehabilitation by the NIHDI. And then I think, well, all those people who are suicidal, they are allowed to stay, can't they, so why can't we? Well, apparently that isn't really taken into consideration."***<br><br>*Female, 43 years*
Open discussion about the death track within treatment trajectory<br><br>***"That one could ask for it, that there was room to discuss it, that there was at least a possibility for other people to engage in it, to assess the urgent need, the despair that you feel at a certain moment in your life: 'I can't do this anymore', 'I can't bear it any longer', 'This is too much', 'This takes too long', 'This is all way too painful'. The fact that, that you can apply for it, that gave me so much peace of mind."***<br><br>Female, 55 years	No discussion of the death track within treatment trajectory talks on death ideation/euthanasia not being included in the existing treatment trajectory<br><br>***"I think it was something of a no go for [name of the treating psychiatrist], that he was like: 'I'm not going down that road. For me it's a subject I don't want to discuss', right? And then I actually started looking for a place where I was able to discuss it and then I switched physicians, didn't I? [...] Dr. [treating psychiatrist] then said: we are ending our therapeutic trajectory now, yes well, we both felt that the therapeutic relationship was depleted."***<br><br>Female B, 43 years
New referrals & treatment approaches<br><br>***"And I was like "Okay, yes, that's fine, I'll get tested". And yes, the testing did indeed reveal the diagnosis of ASS and, well, the thing is that people who are more highly gifted intellectually, especially women, well, they unconsciously learned to camouflage it. But that takes so much energy and that was exactly what made me...***	Referral & further treatment burden<br><br>***"And then they said: 'Look, this [medication] isn't working out, let us try something else?" "Well, yes, of course" I had to rapidly say yes. Uhm, so yes, the former medication was phased out as quickly as possible, and we did a few blood tests. "And yes indeed, one drug has a different impact, its impact differs from***

Because, for example, eating and so on is still a problem for me, but now that I know that I am not able to notice a feeling of 'hunger', that this kind of stimulus does not reach me, just like, uhm, I do not get the notion of 'pain' either. But on the other hand, I am extremely sensitive to medication. So now I know all of that, and, well, okay, at that time I was like, okay, at least we know that now."<br><br>*Female, 33 years*	***one person to another, so let's try something else, he said." And I said, okay, let's try something else, as I'm still willing to consent, I still want to be cooperative, uhm, okay, the new medication dose was increased, and it didn't work out. The side effects were so intense and very burdening me, but for me, the only goal remained to comply with the conditions set by (name advising physician) to get the approval. And then he asked me: "Do you want to try something else? It was then that I said, "Well, no, I don't really need to, because I think I've proven and shown enough that I'm willing to consent and give it a try, so yes, I think of myself that I've done everything that was noted on the to do list. I have done what you asked me to do, I've done my homework, and marked 10 out of 10."***<br>*Female, 51 years*
	Souring patient - physician relationship during the euthanasia trajectory<br><br>***"My (treating therapist) also wrote this in a report. She wrote a report about the fact that I was following behavioural therapy for 2 years now and she also wrote that 'whatever decision she makes, I support her in her decision.'***<br>*(Interviewer: And how did that affect you?)*<br>***Well, I was very happy until…! Then she had a talk with my psychologist, with my general physician and, uh, she also had Dr. (the first advising doctor) on the phone... and, uh, they all said, so all my doctors, they all said that they take me seriously, but that they don't think I'm eligible for euthanasia. And that contradicts the report of my (therapist). She wrote "whatever (...) decides, I'll support her". A month later, she said that to me and I was devastated. She said, "Well yes, I don't think you qualify for euthanasia now". So, I was shocked and I said "You are turning your back on me. First you say that you'd support me and then you say, yes, well, the doctor doesn't know you well enough. And then she said "Well, the diagnosis, we have to be critical about that too and have it examined. And I thought, shit man, I felt so angry inside. You can't play with people like that. They don't realise that sometimes. "***<br><br>Female, 54 years

Table 4 Impact of the euthanasia procedure on APC's Social Life

<table>
<tr><th colspan="2">SOCIAL CIRCLE</th></tr>
<tr><td>Favourable</td><td>Unfavourable</td></tr>
<tr><td>Ability to learn from peers (eg. joined forces to make life more bearable/to see alternative options)

"In the end, you must look at it this way: there are eight or at least seven fellow peers there (in a rehabilitation-oriented group consisting solely of people with a euthanasia request), who all have a wish for euthanasia or a wish for death, and they either have approval or they haven't obtained it yet, or they are in the process of obtaining approval. And they are all people who have tried a ridiculous amount of things during their lifetime. You can't have a greater resource of little tools... Yes, and that is, uhm, what I've heard there sometimes, eh? Yes well, please do try this now or try that and then I think: 'Oh, but that's a great idea, I'm going to try that too, yeah! Or for example, 'I tried this, and it didn't work out for me but maybe it will for you'. So, there you have the largest source of self-help and experiences with therapies and psychiatrists and doctors and everything you want, all together in one single group of fellow peers. Nowhere is that kind of resource so huge."

Female, 33 years</td><td>Not being supported or understood

"No, unfortunately it's not up for discussion, both mum and dad don't want to hear about my wish for euthanasia, so it's difficult. Not only my mother and father, but my whole family also doesn't want to hear about it. I also have uncles and aunts, but they don't want to hear about it, none of them. *(Interviewer: And did you tell them yourself at a certain point or...?* ***Yes, I tried several times, and when I noticed that I couldn't do it verbally, I did it in writing, I did it in every possible way, but they don't want to talk about it.*** *(Interviewer: Is that what they say, 'I don't want to hear about it'?)* ***No, no, they don't even say that, they just run away or they just ignore it."***

Male, 29 years</td></tr>
<tr><td>Empowered to open-up/build new relationships

"And then by chance, in springtime, I think it was February or March, I encountered someone who had become a coach, who also danced with us in (name dancing therapy). But in (name dancing therapy) no talking is allowed during the dance session. *(Interviewer: Ah?)* ***Yes, that is to be able to stay connected to your feelings and, yes, I found out by accident that (dance coach) had to stay at home for 15 years, also due to a (psychiatric disorder). And (dance coach) said "I was against medication for 15 years and at some point, I had to give in to it, but it's often a long search before you find the right meds. And then I asked what did you take then? And (dance coach) gave me the names of the drugs, it was a mix of everything, and (dance coach) still came through. And then (dance coach) studied to become a coach and now (dance coach) works as an independent coach. (dance coach) says: 'you need to start thinking about it and open up to give it a try. So I took the list of named medications to my psychiatrist and gave it a try. Uhm, and I'm also going to a meditation group now, those people have also joined in my life, yes. So, yes, what has left my life is about to get replaced, some new people and new things fill in the places left empty".***

Female, 52 years</td><td>Crumbling relationships

"And I, uhm, it actually ended up with quite a lot of people saying, 'I love you, but I have to let you go'. Yes well, so I don't have any contact with a lot of friends, a lot of people from the past, anymore. *(Interviewer: Okay, but could they just let you go, or have difficulty doing so?)* ***Yes, well, they just didn't understand, or they just went on with their lives, right? I mean in the sense of making their own living, their own home, starting a family, and so on."***

Female, 33 years</td></tr>
<tr><td>Receiving more support in life

"But my daughter has been incredible because she also said, a few days after I had told her about my request for euthanasia, "That must have been terribly difficult for you". I said "yes, it still is terribly difficult". But they didn't say to me "You can't opt for</td><td></td></tr>
</table>

euthanasia" and "you can't…". Afterwards, my daughter said "(name interviewee), I have seen how far you can go in your lows. I love you when you are manic, but now in December, I have seen that being extremely manic is a danger to you, that the urge to kill yourself is so high that you would actually do it." And therefore, she is now determined to stay more alert to the signals, like "Ha (...) you're manic, you're the active mum now." *(Interviewer: Is she going to keep a closer eye on it and try to tone it down?)* ***Yes, she will."***<br><br>*Female, 54 years*	
Opportunity for loved ones to receive support (A)<br><br>***"And euthanasia, well, you can say goodbye, the people who will stay here can be prepared for that moment. I would do anything to achieve that. I wanted them to meet with my psychologist. I wanted them to have guidance during the procedure, yes. Saying goodbye also, I, I, knew who I'd allow to stand at my bedside, yes, I found that, I found that so much more serene than just leaving by surprise."***<br><br>*Female, 47 years*	
	Difficulties with involving and managing interactions with important others<br><br>***"My daughter started crying and said "yes, but that means that, I won't have a mum any more within a year or two". And that was terribly confronting for her. My son said "Yes, then you might as well commit suicide now, than that I have to sit here and wait another two years to see you go, and what are you going to do in the meantime?"*** *(Interviewer: So, 2 different kinds of reactions really?)*<br>***Yes, and yet, a week or two later, my daughter said "Well, for me euthanasia and suicide are the same thing". (Interviewer: Yes?) Yes, and I said "No, I can say goodbye if I opt for euthanasia. And she said "Yes, but in both cases you will be dead. The outcome is death."*** *(Interviewer: But your son had more difficulties with euthanasia?)* ***Yes, yes. He literally said to me, "I'd prefer you to die within the week than in 2 years." He just finds that terrible, another 2 years from now."***<br><br>Female, 54 years
	Comparing own situation with fellow peers<br><br>***"The people I know who have already obtained the approval, for some of them it has been given so quickly, so easily, yes, not quickly, but easily.*** *(Interviewer: You mean the approval?)* ***Yes, so easily given, and what I notice, these people, they don't have it carried out. Like (example in the media), they don't go that far and have it carried out. And one of those people I know, when he came back from the consultation with the approval in his pocket, when he came outside from the consultation with, I don't know which psychiatrist or doctor, and when he went back home, he thought, I don't want to die. (...) - I'm not going to make him identifiable now - he told me that he had only had one conversation with those two physicians, three physicians were mandatory, and in those days,***

	you weren't even supposed to have two (advising) psychiatrists. And they would have told him something like 'we're not going to force you to live' and that he then obtained their approval. *Interviewer: After one conversation?* ***With the first psychiatrist he consulted, I don't know how many conversations he had, but this man still says to me 'they almost threw their approval in my lap'."***<br><br>*Male, 57 years*
